# Perception of length and orientation in dry immersion

**DOI:** 10.3389/fncir.2023.1157228

**Published:** 2023-04-12

**Authors:** Vsevolod Lyakhovetskii, Andrey Chetverikov, Inna Zelenskaya, Elena Tomilovskaya, Valeriia Karpinskaia

**Affiliations:** ^1^Institute for Cognitive Studies, Saint Petersburg State University, Saint Petersburg, Russia; ^2^Donders Centre for Cognitive Neuroimaging, Radboud University, Nijmegen, Netherlands; ^3^Laboratory of Gravitational Physiology of the Sensorimotor System, Institute of Biomedical Problems, Russian Academy of Sciences, Moscow, Russia

**Keywords:** visuomotor processing, motor oblique effect, hypermetria, orientation, dry immersion (DI)

## Abstract

**Introduction:**

How does gravity (or lack thereof) affect sensory-motor processing? We analyze sensorimotor estimation dynamics for line segments with varying direction (orientation) in a 7-day dry immersion (DI), a ground-based model of gravitational unloading.

**Methods:**

The measurements were carried out before the start of the DI, on the first, third, fifth and seventh days of the DI, and after its completion. At the memorization stage, the volunteers led the leading hand along the visible segment on a touchscreen display, and at the reproduction stage they repeated this movement on an empty screen. A control group followed the same procedure without DI.

**Results:**

Both in the DI and control groups, when memorizing, the overall error in estimating the lengths and directions of the segments was small and did not have pronounced dynamics; when reproducing, an oblique effect (higher variability of responses to oblique orientations compared to cardinal ones) was obtained. We then separated biases (systematic error) and uncertainty (random error) in subjects’ responses. At the same time, two opposite trends were more pronounced in the DI group during the DI. On the one hand the cardinal bias (a repulsion of orientation estimates away from cardinal axes) and, to a small extent, the variability of direction estimates decreased. On the other hand, the overestimation bias in length estimates increased.

**Discussion:**

Such error pattern strongly supports the hypotheses of the vector encoding, in which the direction and length of the planned movement are encoded independently of each other when the DI disrupts primarily the movement length encoding.

## 1. Introduction

The problems of space travel have been in focus of scientists for several 100 years. Kepler (1634) proposed to choose men with good health and sedate them to prevent damage of start acceleration. Tsiolkovsky (1920), describing awake astronauts, predicted that they would be subject to phantasmagorical sensations due to a loss of body weight sensation. Indeed, empirical studies show that adaptation to weightlessness affects how astronauts perceive the world (Paloski et al., 2008; Arshad and Ferré, 2022). For example, Kornilova (1997) showed that such adaptation creates spatial orientational illusions that are illusions pertaining to subjects position or illusions of self- and surround-motion. Some studies show that the mental representation of the vertical dimension of objects is altered in space (Clément, 2007). However, despite a long-time interest in the topic of perceptual distortions in space, there are relatively few studies investigating how they arise.

The onset of changes varies for different visual phenomena. Clément et al. (2012) showed that the strength of inverted-T illusion (overestimation of the length of a vertical line relative to a horizontal line of the same length) measured in an adjustment task lowered significantly only on the fifth month of spaceflight, while the ratio of vertical to horizontal line during drawing a cross or square diminished earlier, after 1 month of spaceflight. That is, two similar perceptual effects related to perception of horizontal and vertical lines, but measured differently, were both affected by weightlessness but with varying onsets of changes. Such difference in dynamics of perception of vertical dimension in these two tasks may be due to more involvement of the dorsal stream in performance of the latter one (Karpinskaia et al., 2022). This highlights the importance of studying the dynamics of visual perception during adaptation to weightlessness.

In the current project, we aimed to study the dynamics of length and orientation perception in a simulated weightlessness using the dry immersion (DI). DI is one of the ground-based models of spaceflight allowing to study the effects of space flight in a well-controlled environment. During DI, the volunteer is laying on the rubber textile in the bath filled with warm water (Tomilovskaya et al., 2019). The vestibular system activity changes due to elimination of the support and minimization of proprioceptive afferentation.

Orientation and length are well-studied low-level visual features, important for downstream visual processing in the brain. However, there are only a few studies analyzing how processing of these features is affected by weightlessness (Lipshits and McIntyre, 1999; Lipshits et al., 2005; McIntyre and Lipshits, 2008). Interestingly, orientation perception is characterized by systematic anisotropies: the precision of estimates is lower for oblique orientations compared to cardinal (the oblique effect) while at the same time the responses are biased away from the cardinal orientations toward oblique ones (the cardinal bias; see Appelle, 1972; Tomassini et al., 2010; Girshick et al., 2011; Wei and Stocker, 2017). Despite the absence of a gravitational vertical, this pattern of responses persists at the end of the first month of spaceflight during performing different types of adjustment tasks (visual and haptic, McIntyre and Lipshits, 2008), that is constant and variable errors reflecting bias and SD of orientation estimation do not change.

However, previous studies used an adjustment task with a joystick even in a haptic domain, and the use of such an instrument activates to a greater extent the ventral stream (Ferrari et al., 2005) presumably less affected by gravity change (Karpinskaia et al., 2022). Considering that, firstly, DI influences the hand movements similarly to real microgravity (Kornilova et al., 2011), and, secondly, the hand movements turned out to be more sensitive to anisotropy of human perception during spaceflight than the adjustment procedure (Clément et al., 2012), we chose the motor version of oriented segments task (Smyrnis et al., 2007; Pantes et al., 2009). The use of the motor task allows also to register simultaneously the length and the orientation of a given segment. We hypothesized that DI would gradually alter the perception of segment length and/or its orientation.

## 2. Materials and methods

The DI group consisted of 10 male volunteers (30.9 ± 4.6 years) who were in a 7-day DI. The control group consisted of 22 volunteers (5 males and 17 females, 31.6 ± 7.6 years). In the DI group, the measurements were performed before immersion (D0), on the 1st (DI1), 3rd (DI3), 5th (DI5), and 7th (DI7) day of DI, and after its end (R + 1). The subjects were admitted to participate in the experiment by a medical expert commission and signed an Informed Consent to participate in the study in accordance with the provisions of the Helsinki Declaration of Human Rights. The research procedures were preliminary reviewed and approved by the Commission on Biomedical Ethics of the Institute of Biomedical Problems of the Russian Academy of Sciences (Protocol No. 1 of Sept. 09, 2021). To mimic the time course of measurements in DI, the control group was studied on the 1st (D0), 2nd (D1), 4th (D3), 6th (D5), and 8th (D7) days.

**FIGURE 1:**
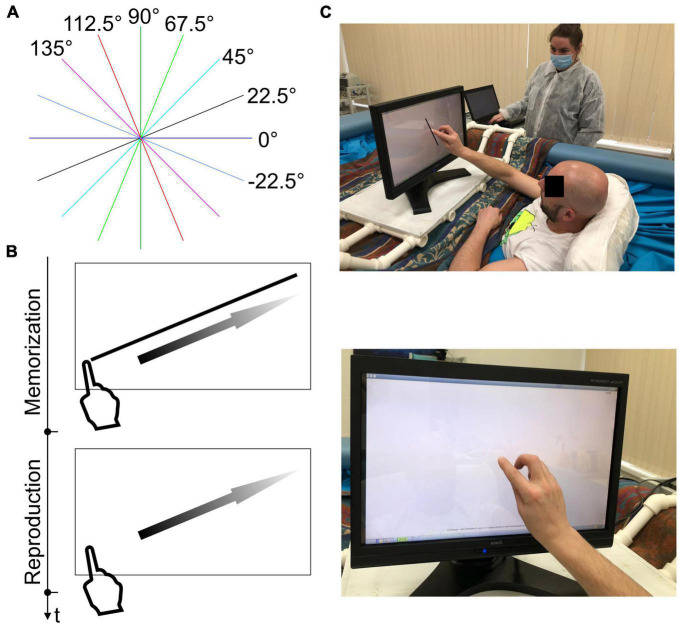
Experimental design. **(A)** Stimuli material. One segment directed at the specified angle was presented in each probe (segments are colored for demonstration purposes). **(B)** In the memorization stage, the volunteer moved the index finger of his dominant hand over the segment. Then the experimenter pressed the button on the keyboard, the stimulus disappeared, and the volunteer reproduced the memorized parameters of the given segment at the same location on an empty screen. **(C)** Memorization and reproduction stages during dry immersion.

The black segments oriented at −22.5°, 0°, 22.5°, 45°, 67.5°, 90°, 112.5°, and 135° to the horizontal (Figure 1A) were presented on a white background in random order in the center of the volunteer’s visual field, 4 presentations for each orientation. The set of orientations was similar to that used in Lipshits and McIntyre (1999). The centers of the segments were in the center of the volunteer’s visual field. The segments started from different points of the visual field, so the volunteer didn’t get used to the stable initial point of his/her movement.

In the DI group, stimuli were presented on the LCD optical multi-touch monitor (IIYAMA Prolite T2252MTS, iiyama, Tokyo, Japan) with a viewable area of 476 by 268 mm [gamma value of 2.2, color temperature (white point) of 6500K, and luminance during touch of 200 cd/m^2^]. The size of the presented segment was 10 cm. A notebook (Acer Spin SP111-34N, Xizhi, Taiwan) with a touchscreen having a viewable area of 260 by 143 mm [gamma value of 2.2, color temperature (white point) of 6500K, and luminance during touch of 250 cd/m^2^] was used for the longitudinal control group study. The size of the presented segment was 5.4 cm. For both groups, the screen resolution was 1920 × 1080.

The participant was in a semi-sitting position (Figure 1C) in a bath (during DI) or on the couch in front of the monitor, at a distance of 60−80 cm to establish the comfort hand movement over the screen surface. Their task was to reproduce the lengths and directions of the segments with the dominant (in our groups, right) hand. First, at the memorization stage, the volunteer moved the index finger of the dominant hand from left to right (from top to bottom in the case of a vertical segment) along the visible segment (Figures 1B, C). When the subject lifted his hand from the screen, the experimenter pressed a button on the keyboard, the segment disappeared, and the volunteer reproduced the memorized parameters of the given segment at the same location on an empty screen immediately. The experiment was performed without feedback.

The coordinates of start and end points of the hand movements were determined on the touch screen. Based on these coordinates, the estimated length of the segment was calculated as the Euclidean distance between the start and end points of the hand movement, and its direction was determined. The segment length estimation error was determined as the difference between the segment length determined by the volunteer and the known segment length, the segment direction estimation error as the difference between the segment direction determined by the volunteer and the known segment direction. To calculate the cardinal bias, we analyzed the errors for orientations between cardinal and oblique ones (−22.5°, 22.5°, 67.5°, and 112.5°) with the sign for orientations where a negative bias is expected (−22.5° and 67.5°). The bias of length estimation for segments of non-cardinal orientations was calculated as the mean non-cardinal segment length estimation error.

We analyzed separately the obtained datasets for two groups. Approximately a quarter of studied datasets were not distributed normally by D’Agostino-Pearson criterion. Thus the significance of differences between the obtained values was assessed using the paired samples Wilcoxon test with Bonferroni correction at *p* < 0.05. Data are presented as mean ± standard deviation.

## 3. Results

**FIGURE 2:**
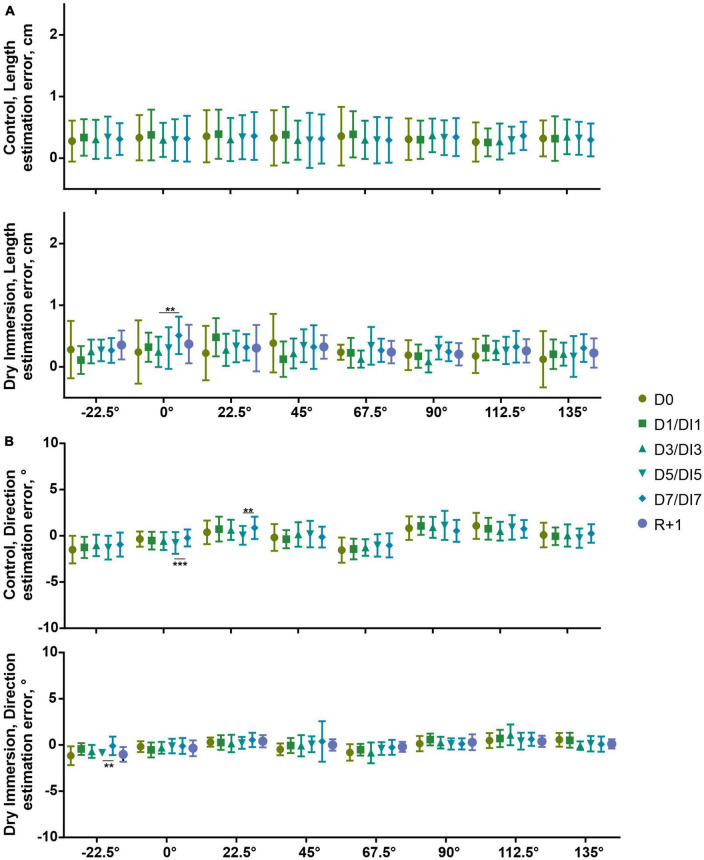
Estimation of length **(A)** and direction **(B)** of segments of different orientation at memorization stage in control (*n* = 22) and DI (*n* = 10) groups. Abscissa-orientation of given segment. D0–1st day of measurements in control and DI groups. D1, D3, D5, and D7–2nd, 4th, 6th, and 7th day of measurements in the control group, respectively. These days correspond to DI1, DI3, DI5, and DI7–1st, 3rd, 5th, and 7th day of DI. R + 1–the measurement performed 1 day after the end of the DI procedure. Mean ± SD. **p < 0.01, ***p < 0.001.

At the memorization stage, when the participant moved the hand over the visible segment, the errors of estimation of both length and orientation were considerably small (Figure 2). Both groups overestimated the length of the segment (0.32 ± 0.28 cm, U(22) = 245, *p* < 0.001 and 0.26 ± 0.14 cm, U(10) = 54, *p* < 0.01, for control and DI group, respectively), and this overestimation didn’t depend on the day of measurement (with the unique exception of the increase of overestimation of the horizontal segment in DI7 relative to DI3 in the DI group, *p* < 0.01) (Figure 2A). The error of orientation didn’t also have any pronounced dynamics. In the control group, this error diminished in D7 relative to D5 for the horizontal segment (*p* < 0.001), and increased in D7 relative to D5 for the 22.5° segment (*p* < 0.01). In the DI group, the orientation error of the −22.5° segment diminished in DI7 relative to the DI5 (*p* < 0.01) (Figure 2B).

**FIGURE 3:**
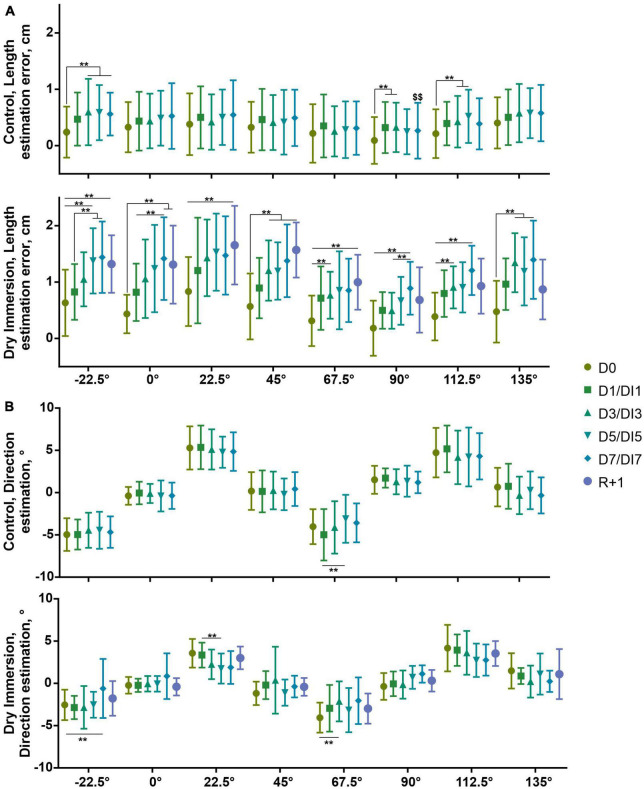
Estimation of length **(A)** and direction **(B)** of segments of different orientation at reproduction stage in control (*n* = 22) and DI (*n* = 10) groups. Abscissa–orientation of given segment. D0–1st day of measurements in control and DI groups. D1, D3, D5, and D7–2nd, 4th, 6th, and 7th day of measurements in the control group, respectively. These days correspond to DI1, DI3, DI5, and DI7–1st, 3rd, 5th, and 7th day of DI. R + 1–the measurement performed 1 day after the end of the DI procedure. Mean ± SD. $$*p* < 0.01 in relation to horizontal segment, **p < 0.01 in relation to the estimation of the segment of the same orientation on other days.

At the reproduction stage when the participant moved the hand over the empty screen the length and orientation errors possessed different dynamics (Figure 3). Both groups overestimated the length of the segment (0.41 ± 0.39 cm, U(22) = 234, *p* < 0.001, and 0.98 ± 0.39 cm, U(10) = 55, *p* < 0.01, for control and DI group, respectively) (Figure 3A). In the control group, the overestimation increased in relation to D0 only for the −22.5° segment and for the 112.5° segment (*p* < 0.01). The estimated length of the horizontal segment is significantly larger than the length of the vertical segment in the D7 only (*p* < 0.01).

In the DI group, the overestimation dynamics is more pronounced. The mean linear trend of increasing overestimation was 0.10 ± 0.02 in DI group vs. 0.02 ± 0.01 in control group (W(8) = 36, *p* < 0.01). The overestimation increased for all orientations of the segment relative to D0 (all *p*s < 0.01). The overestimation is increased not only in comparison with D0 but for −22.5°, and 0° segments in comparison to DI1, and for the 90° segment in comparison to DI3 (all *p*s < 0.01).

In both groups, we observed the cardinal bias (Smyrnis et al., 2007) during all study (Figure 3B). The volunteers underestimated the orientation of −22.5°, and 67.5° segments (4.3 ± 1.4°, U(22) = 253, *p* < 0.001, and 2.5 ± 1.3°, U(10) = 55, *p* < 0.01, for control and DI group, respectively). These orientations were repulsed from cardinal axes and were attracted to −45° and 45°, respectively. Similarly, the volunteers overestimated the orientation of 22.5°, and 112.5° segments (4.8 ± 2.0°, U(22) = 253, *p* < 0.001, and 3.1 ± 0.8°, U(10) = 55, *p* < 0.01, for control and DI group, respectively). These orientations were repulsed from cardinal axes and were attracted to 45° and −45°, respectively. The direction estimation errors decreased. In the control group, the direction of the 67.5° segment was estimated more accurately; in the DI group the directions of the −22.5°, 67.5°, and 22.5° segments were estimated more accurately (all *p*s < 0.01).

**FIGURE 4:**
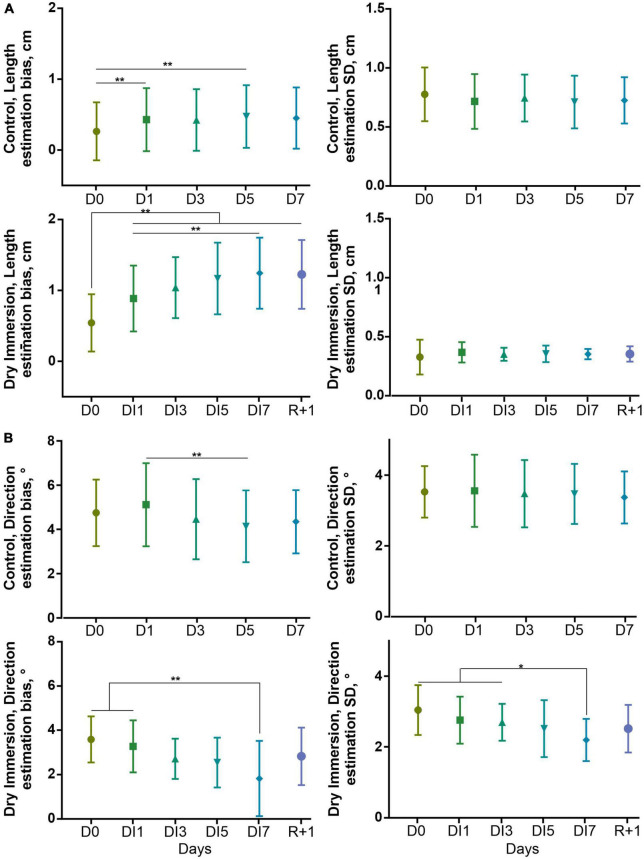
Bias and SD of estimation of length (all orientations), **(A)** and direction (non-cardinal segments), **(B)** at reproduction stage in control (*n* = 22) and DI (*n* = 10) groups. The bias of length estimation was calculated as mean non-cardinal segment length estimation error. The directional bias was calculated as (O_22_._5_+O_112_._5_–O_–22_._5_–O_67_._5_)/4 where O is a direction error of given participants for given direction. D0–1st day of measurements in control and DI groups. D1, D3, D5, and D7–2nd, 4th, 6th, and 7th day of measurements in the control group, respectively. These days correspond to DI1, DI3, DI5, and DI7–1st, 3rd, 5th, and 7th day of DI. R + 1–the measurement performed 1 day after the end of the DI procedure. Mean ± SD. **p < 0.01, *p < 0.05.

The bias and SD of errors for non-cardinal orientations are summarized in Figure 4. In the control group, the overestimation of segment length is increased in D1, and D5 relative to D0 (0.43 ± 0.44 cm vs. 0.26 ± 0.14 cm, W(22) = 165, *p* < 0.01, and 0.47 ± 0.44 cm vs. 0.26 ± 0.14 cm, W(22) = 163, *p* < 0.01, respectively). In the DI group the overestimation of segment length is increased to a greater extent: in DI1, DI3, DI5, DI7, and R + 1 relative to D0 (0.89 ± 0.46 cm vs. 0.54 ± 0.40 cm, W(10) = 53, *p* < 0.01, 1.04 ± 0.43 cm vs. 0.54 ± 0.40 cm, W(10) = 53, *p* < 0.01, 1.17 ± 0.50 cm vs. 0.54 ± 0.40 cm, W(10) = 53, *p* < 0.01, 1.24 ± 0.50 cm vs. 0.54 ± 0.40 cm, W(10) = 55, *p* < 0.01, and 1.23 ± 0.48 cm vs. 0.54 ± 0.40 cm, W(10) = 43, *p* < 0.01, respectively); in DI7 relative to DI1 (0.89 ± 0.46 cm vs. 1.24 ± 0.50 cm, W(10) = 49, *p* < 0.01). In the control group, the direction error is decreased in D5 relative to D1 (5.1 ± 1.9° vs. 4.1 ± 1.6°, W(22) = 191, *p* < 0.01). In the DI group, again, the decrease of direction error is more pronounced (DI7 relative to D0, and DI1: 1.8 ± 1.7° vs. 3.6 ± 1.0°, W(10) = 53, *p* < 0.01, and 1.8 ± 1.7° vs. 3.3 ± 1.2°, W(10) = 55, *p* < 0.01, respectively) and accompanied by trend in decrease of its SD (DI7 relative to D0, DI1, and DI3: 2.2 ± 0.6° vs. 3.0 ± 0.7°, W(10) = 43, *p* < 0.05, 2.2 ± 0.6° vs. 2.8 ± 0.7°, W(10) = 43, *p* < 0.05, and 2.2 ± 0.6° vs. 2.7 ± 0.5°, W(10) = 47, *p* < 0.05, respectively).

## 4. Discussion

Here we replicated the finding of the motor oblique effect and cardinal biases (Baud-Bovy and Viviani, 2004; Smyrnis et al., 2007; Pantes et al., 2009), that is the memorized segment orientations have higher variability at oblique orientations compared to cardinal and are systematically shifted away from the cardinal axes. Thus, this effect is persisted in DI as well as in space (McIntyre and Lipshits, 2008).

In our study, the segment length is overestimated in both groups, both for memorization and reproduction stages. This is in contrast to previous studies showing that elimination of visual feedback from the moving arm leads to hypometric (having smaller than required movement amplitude) pointing movements (Bock and Eckmiller, 1986) or that hiding the memorized target eliminates hypermetria (higher than required movement amplitude) induced by previous experimental manipulations (Avraham et al., 2019). It seems that the overestimation of target position or the movement length is more typical for arm movements irrespective of visual feedback (Baud-Bovy and Viviani, 2004; Pantes et al., 2009; Avraham et al., 2017, 2019). Simulated or real microgravity also leads to hypermetria. Hypermetric cyclic arm movements were observed in 6 h and 5 days DI (Lyakhovetskii et al., 2022); hypometric pointing arm movements became hypermetric during spaceflight (Tomilovskaya and Kozlovskaya, 2012).

The vertical length of hand-drawn objects [ellipses (Gurfinkel et al., 1993), letters (Clément et al., 1987), cube edges (Lathan et al., 2000)] is decreased in space. Interestingly, we saw a significant difference in reproduction of length of horizontal and vertical segments at D7 for the control group only (Figure 3A). Speculatively, the dynamics of change of vertical to horizontal ratio observed in space (Lathan et al., 2000; Clément et al., 2012) may be due to the effect of prolonged training and not to microgravity by itself. Thus, our study underlines the importance of use of the corresponding control group to investigate the prolonged perceptual effects.

Stimulus configuration may affect the motor responses even with visual feedback though such influence is lower compared to when the visual feedback is absent. The arrows of Muller-Lyer illusion affect the movement amplitude even when the stimulus is clearly visible (Gentilucci et al., 1996); the circles of Ebbinghaus illusion influence the movement time (van Donkelaar, 1999); the Ponzo and Muller-Lyer illusions also modulate the motor responses during the course of 5-day DI (Sosnina et al., 2019). In our case, the effects observed in the memorization phase are mainly similar to those received in the reproduction phase but they have lower magnitude.

Two broad classes of hypotheses suggest the positional (desired position is coded) or vector (direction and distance are coded) internal representations of hand movement targets (Kim et al., 2021). An analysis of errors distribution is one way to explore the internal representation used (Hudson and Landy, 2012). If the subject uses a given coding scheme during repeated trials, this encoding may become more precise during training (van der Graaff et al., 2017). Previous studies suggest that length estimation is more error-prone and more variable relative to estimation of direction, indicating vector-based encoding. Hand movements drift from hypometric to hypermetric while direction bias doesn’t drift consistently during the 25 min visuomanual pointing (Vindras and Viviani, 1998). Adaptation to gain change influencing movement length (an altered relationship between distance moved on the screen and the distance moved on the tablet) is faster and more complete than adaptation to space rotation influencing direction errors (Krakauer et al., 2000). During the time course of our study in both groups the direction error of the reproduced segment is decreased while the overestimation of segment length is increased. Such a complex pattern of errors strongly supports the hypothesis on the vector encoding of movement goals, which is typical for the movements of the dominant hand, when the direction and length of the planned movement are encoded independently of each other (Gordon et al., 1994; Vindras et al., 2005). The behavioral data find support in single-cell electrophysiological recording. The neuronal discharge patterns registered in monkey primary motor and pre-motor cortex are in favor of independent amplitude and directional coding. This neuronal activity relates primarily to direction encoding; the movement amplitude is coded after the direction of movement is chosen (Riehle and Requin, 1989). Thus, the proposed experimental design may be also useful for studying the encoding scheme of hand movements.

Observed opposite changes of two types of errors are more pronounced in the DI group. It’s possible that DI volunteers participating in a complex scientific experiment might be more motivated and have higher accuracy relative to the control group. This might be true for orientation errors, however, their length overestimation bias is expressed to a greater degree. One possible explanation is that according to H. Jackson dissolution theory (Meares, 1999) the properties of perception emerging late during development are the most fragile. In fact, the overestimation of distance is appeared later in development than correct estimation of orientation: overshooting of target location is absent in young children aged 6−7, observed in the adult group, and reaches maximum in the older children group aged 10−11, while the direction error is the same for different age groups (Pantes et al., 2009). The estimation of orientation is immune to Parkinson’s disease: patients exhibit hypometria without any direction bias (Desmurget et al., 2003). Thus, DI influence onto the vestibular system might affect primarily the length estimation. Another possible explanation is based on the different roles of hemispheres on movement control. The left hemisphere of right-handers has a greater role in dynamic control of movement trajectory relative to the right hemisphere (Haaland et al., 2004). An exposure to DI caused a sharp decrease in the left hemisphere activity (Kirenskaya et al., 2006) that presumably may affect the movement length.

## 5. Conclusion

In summary, the analysis of the dynamics of the sensorimotor estimation of the lengths and directions of segments of various orientations by the leading hand in DI was carried out in comparison with the control group. We obtained here four main findings. First, the segment length is overestimated in both groups, both for memorization and reproduction stages. Second, the direction of reproduced segments of non-cardinal orientation is repulsed from cardinal axes. Third, the directional error of the reproduced segment is decreased while the overestimation of segment length is increased during study in both groups. Fourth, such opposite changes of two types of errors are more pronounced in the DI group. To conclude, even the perception of such simple objects as oriented line segments is modulated by the DI.

## 6. Limitations

The DI group consisted of men only while the control group consisted of men and women. The measurements in two groups were performed on the sensor monitors of different sizes. Though the rectangular frame size doesn’t influence the estimation of length (Gavilán et al., 2017) and orientation (Zoccolotti et al., 1992), and sex difference in the perception of orientation (Brabyn and McGuinness, 1979) and inverted-T illusion (Brosvic et al., 2002) are absent, we limited our interest by the potential dynamics of DI influence onto the parameters studied. As it can be seen from the presented results, the observed changes occurred at the beginning of the study, in relation to the first measurements. Therefore, to simplify the recruitment of the control group, measurements in it were carried out for 8 days (and not for 9 days as in the DI group).

## Data availability statement

The data from the study reported in this article is available at https://dx.doi.org/10.17605/OSF.IO/3QCJ9.

## Ethics statement

The studies involving human participants were reviewed and approved by the Commission on Biomedical Ethics of the Institute of Biomedical Problems of the Russian Academy of Sciences (Protocol No. 1 of Sept. 09, 2021). The patients/participants provided their written informed consent to participate in this study.

## Author contributions

AC and VL conceived the experiments and analyzed the data. VL, VK, and AC wrote the manuscript. VL, AC, VK, IZ, and ET edited the manuscript. IZ performed the research. VK and ET supervised the study. All authors contributed to the article and approved the submitted version.
